# Giant nonfunctioning adrenal tumors: two case reports and review of the literature

**DOI:** 10.1186/s13256-018-1876-8

**Published:** 2018-11-10

**Authors:** Chatzoulis George, Passos Ioannis, Bakaloudi Dimitra-Rafailia, Giannakidis Dimitrios, Koumpoulas Alexandros, Ioannidis Konstantinos, Tsifountoudis Ioannis, Pappas Dimitrios, Spyridopoulos Panagiotis

**Affiliations:** A’ Department of Surgery, 424 General Military Hospital of Thessaloniki, Agiou Nikolaou 42, 55132, Kalamaria, Thessaloniki, Greece

**Keywords:** Nonfunctioning, Adrenal tumors, Adrenocortical carcinoma (ACC), Adrenalectomy, Case report, Hormone secretion

## Abstract

**Background:**

There are an estimated 1–2 cases per million per year of adrenocortical carcinoma in the USA. It represents a rare and aggressive malignancy; it is the second most aggressive endocrine malignant disease after anaplastic thyroid carcinoma. Non-secretory adrenal masses are diagnosed late due to a mass effect or metastatic disease or found incidentally (adrenal incidentalomas).

**Case presentation:**

The first case report describes a 39-year-old Greek woman who presented to our department with complaints of repeated symptoms of flatulence and epigastric discomfort over a few months. The second case report is about a 67-year-old Greek woman who presented to our department after being evaluated for fatigue, mass effect, and epigastric discomfort. Both of them were diagnosed as having a nonfunctioning adrenocortical carcinoma and underwent open adrenalectomy.

**Conclusions:**

Approximately 60% of patients with adrenocortical carcinoma present with symptoms and signs of hormonal secretion. Our cases’ adrenocortical carcinomas were not functional. Hormone secretion is not a discriminating feature between benign and malignant adrenocortical masses. The silent clinical nature of nonfunctioning adrenocortical carcinoma results in late diagnosis, while the majority of patients present with locally advanced and/or metastatic disease.

Adrenocortical carcinoma is a rare endocrine tumor with a poor prognosis that can be diagnostically challenging and demands high clinical suspicion. The work-up for adrenal masses must include determination of whether the mass is functioning or nonfunctioning and whether it is benign or malignant.

## Background

There are an estimated 1–2 cases per million per year of adrenocortical carcinoma (ACC) in the USA [1]. ACC represents a rare and aggressive malignancy [1]; it is the second most aggressive endocrine malignant disease after anaplastic thyroid carcinoma. ACC is more common in the female population; it has a bimodal age distribution of 5 to 20 years and 40 to 50 years [2]. Out of all the adrenal masses, 60% are hyper-functioning (hormone-secreting) and the rest, 40%, are nonfunctioning (non-hormone secreting) [3].

Functional ACC can clinically manifest early with virilization, feminization, or Cushing’s syndrome, while non-secretory adrenal masses are diagnosed late and incidentally (adrenal incidentalomas) due to a mass effect or metastatic disease [4]. Nonfunctioning ACC is correlated with a poor prognosis due to the late diagnosis, local invasion, or recurrence and distant metastases [1, 3, 4].

On the other hand, adrenal cysts are usually benign lesions with a discovery at autopsy range of 0.064–0.18%, in which adrenal pseudocysts account for an extremely small amount [4].

We present the cases of two women who were referred to our Department of surgery and were diagnosed as having a large nonfunctioning ACC. Both women underwent open adrenalectomy.

## Case presentation

### Case 1

A 39-year-old Greek woman, a nurse in our military hospital, presented to our department with repeated symptoms of flatulence and epigastric discomfort over a few months. Her past medical, social, environmental, and family history was unremarkable for any illness or causative factor. She was not on any medication, she did not smoke tobacco or consume alcohol, and she was afebrile at the time of admission. Her neurological examination was normal; her blood pressure was 126/84 mmHg, her pulses were regular at 75–80 beats/minute, and her temperature was 36.8 °C. Laboratory testing revealed the following results that are shown in Table 1: a rise in erythrocyte sedimentation rate (ESR) and C-reactive protein (CRP) inflammation markers as well as a rise in her lactate dehydrogenase (LDH) tissue necrosis index (Table 1).

**Table 1:** Laboratory testing for case report 1

Test	Result	Reference range
Hematocrit (%)	34	37–47
WBC (white blood cells) × 10^9^/L	11,100	4–11,000
PLT (platelet count) × 10^3^/μL	503	142–450
ESR (mm)	67	< 20
Glucose (mg/dl)	112	70–105
Urea (mg/dl)	19	20–45
Creatinine (mg/dl)	0.6	0.72–1.25
Uric acid (mg/dl)	3.3	3–7.2
γGT (U/L)	67	9–36
CPK (U/L)	22	25–110
LDH (lactate dehydrogenase; U/L)	418	125–220
CRP (C-reactive protein; mg/dl)	6.8	< 0.5

*CPK* creatine phosphokinase, *ESR* erythrocyte sedimentation rate, *γGT* gamma-glutamyltransferase

**Fig. 1:**
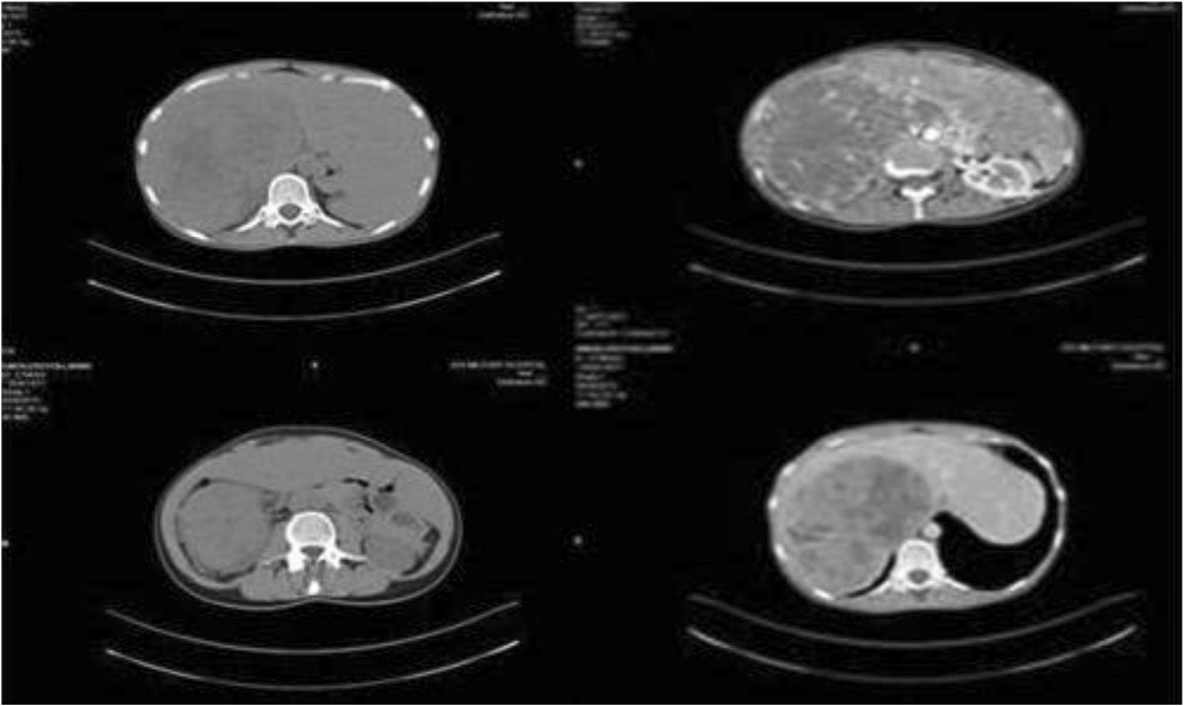
Computed tomography appearance of giant adrenocortical carcinoma

**Fig. 2:**
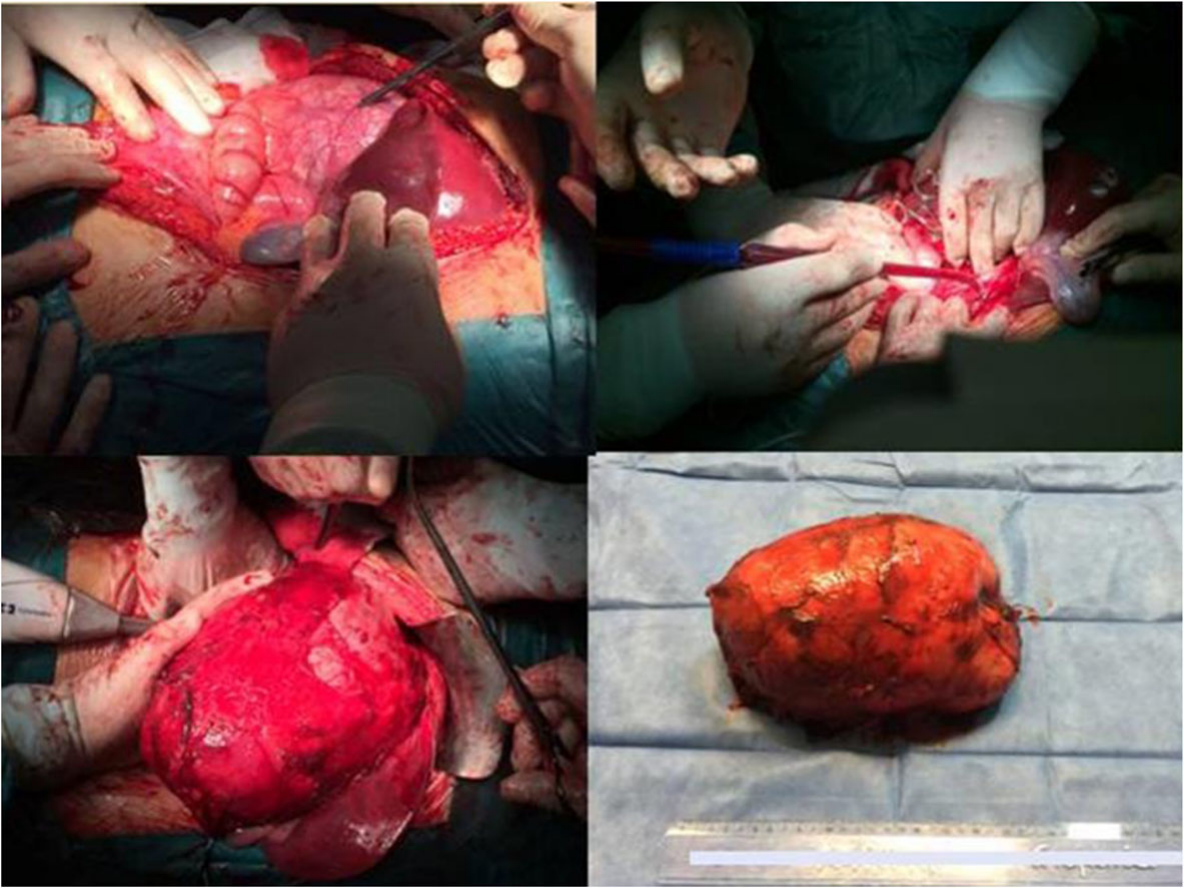
Intraoperative image of giant adrenocortical carcinoma with displacement of right hepatic lobe to the left and vena cava close to the anterior abdominal wall

A computed tomography (CT) scan showed a large mass measuring approximately 24 cm in its greatest dimension (23.7 cm × 16.5 cm × 11.5 cm) that originated from her right adrenal gland and occupied her right abdomen, while compressing her right hepatic lobe and her inferior vena cava, and it was in contact with the right perirenal fascia of Gerota (Figs. 1 and 2).

A functional adrenal work-up was performed and included: measurement of serum aldosterone, potassium, renin, and adrenocorticotrophic hormone levels; a dexamethasone suppression test; and measurement of 24-hour urinary metanephrine levels. All results were within the reference ranges. A fine-needle core biopsy revealed ACC.

A metastatic work-up included CT scans of her head and chest and a bone scan and they were negative for metastases. During laparotomy the giant tumor was removed completely with its own capsule, without the need for excision of adherent organs as there were no infiltrations.

**Fig. 3:**
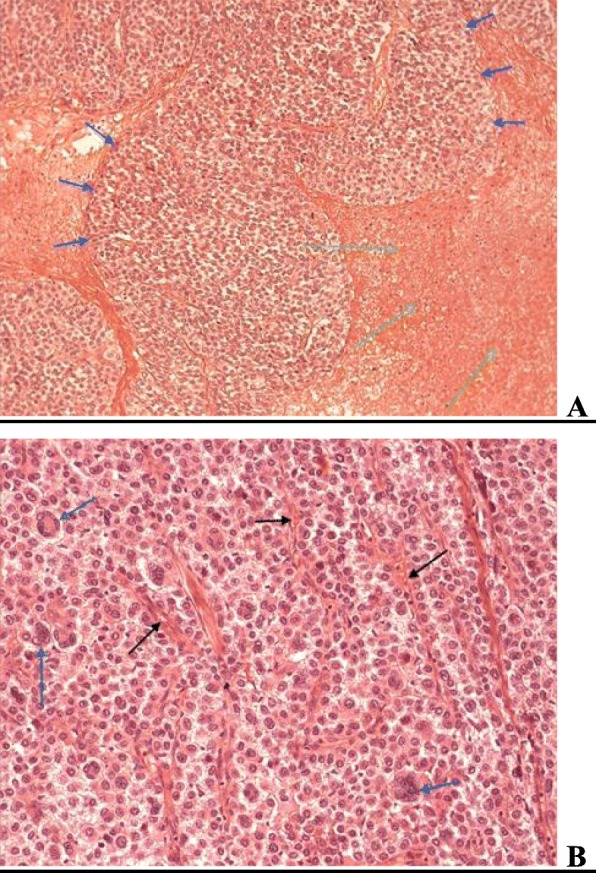
Histological examination. **a** Anastomotic islets (*blue arrows*) of relatively homogeneous neoplastic cells with areas of necrosis and hemorrhagic elements (*green arrows*) with a geographical distribution (hematoxylin and eosin × 20). **b** Part of the tumor, where the neoplastic cells show nuclear pleomorphism; a few of them appear with multiple nuclei (*blue arrows*). Thin capillaries are evident between neoplastic cells (*black arrows*). Hematoxylin and eosin × 20

Postoperative pathology results confirmed the diagnosis of ACC and no further adjuvant treatment was applied to our patient (Fig. 3). Her postoperative course has been uneventful for 1.5 years.

### Case 2

A 67-year-old Greek woman, a retired high-school teacher, presented to our department after an evaluation for fatigue, mass effect, epigastric discomfort in liver cirrhosis, and hypothyroidism. Her past medical history was also remarkable for arterial hypertension. She was on double anti-hypertensive medication and she was also receiving levothyroxine 100 μG once daily. She was a heavy tobacco smoker (>1pack/day) for 35 years and a social alcohol consumer. She was afebrile at the time of admission. Her neurological examination was normal; her blood pressure was 145/97 mmHg, her pulses were 95 beats/minute, and her temperature was 36.4 °C. Her mother died from breast cancer.

**Fig. 4:**
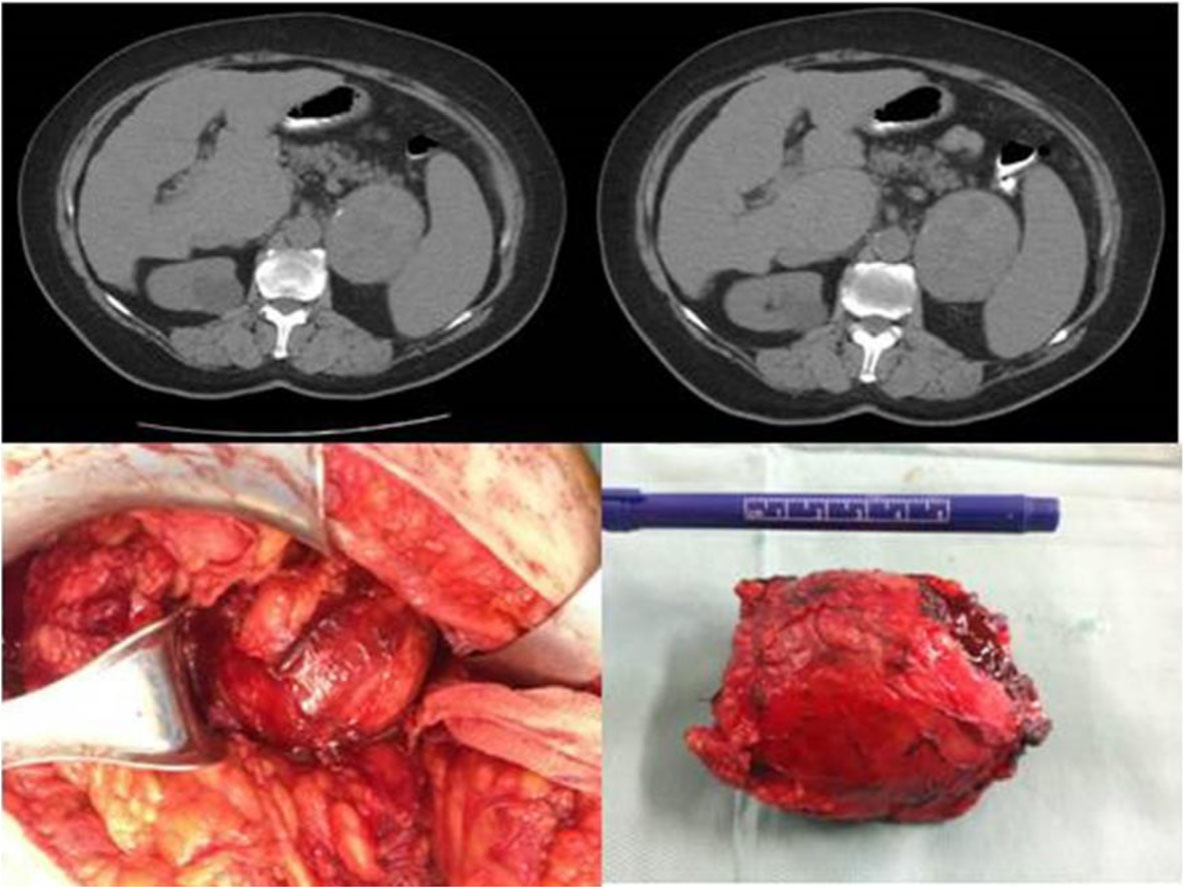
Computed tomography and intraoperative findings of left adrenal pseudocyst

Laboratory testing revealed results that are shown in Table 2. A CT scan revealed a large invasive mass in the anatomical area of ​​her left adrenal gland, well circumscribed, measuring 7 × 7 × 9 cm; it extended to the upper pole of her left kidney and the inner hilum of her spleen without infiltration of the above organs, which showed marked heterogeneous enhancement after intravenous infusion of a contrast agent, which posed a differential diagnosis problem with possible pheochromocytoma (Fig. 4).

**Table 2:** Laboratory testing for case report 2

Test	Result	Reference range
Hematocrit (%)	40.3	37–47
WBC (white blood cells) × 10^9^/L	5600	4–11,000
PLT (platelet count) ×10^3^/μL	145	142–450
ESR (mm)	48	< 20
Glucose (mg/dl)	117	70–105
Urea (mg/dl)	33	20–45
Creatinine (mg/dl)	0.88	0.72–1.25
Uric acid (mg/dl)	7.5	3–7.2
γGT (U/L)	39	9–36
CPK (U/L)	35	25–200
LDH (lactate dehydrogenase; U/L)	276	125–220
CRP (C-reactive protein; mg/dl)	0.6	< 0.5

*CPK* creatine phosphokinase, *ESR* erythrocyte sedimentation rate, *γGT* gamma-glutamyltransferase

**Fig. 5:**
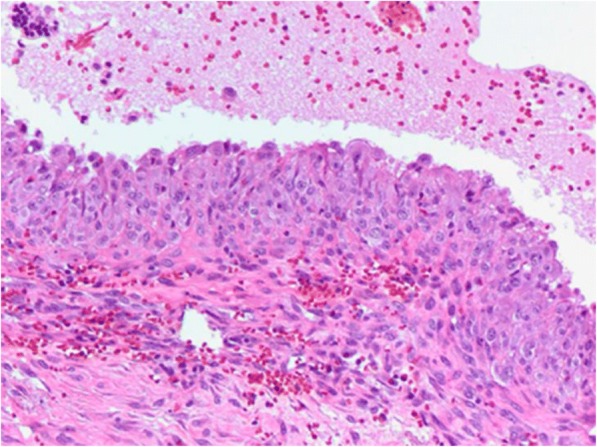
Histological examination: single-stranded cyst with fibrous wall, without epithelial lining, with adherent tissue of adrenal tissue, confirming the diagnosis of adrenal pseudocyst. Hematoxylin and eosin × 20

Further laboratory testing of post-prandial plasma cortisol and plasma testosterone levels gave normal results, mimicking a nonfunctional left ACC. Elective open adrenalectomy was scheduled without any complications and the postoperative pathology record was consistent with a pseudocyst, without evidence of malignancy (Figs. 4 and 5). She has had an uneventful course 1 year postoperatively.

## Discussion

The overall 5-year survival rate of ACC ranges from 16 to 44% [4]. However, a more recent study recorded a 5-year overall survival rate that reached 60% and concerned patients with a 34% range of synchronous metastatic disease. Positive prognostic factors that could improve overall survival are early-stage disease in the absence of lymph node and distant metastases, an age of < 40 years, and negative resection margins (Ro), as in our case [1, 4].

On the other hand, adrenal pseudocysts are cystic lesions surrounded by a fibrous tissue wall with an absence of a recognizable epithelial or endothelial lining layer that characterizes true cysts. Adrenal pseudocysts are associated with malignant ACC in 7% of cases [5].

Approximately 60% of patients with ACC present with symptoms and signs of hormonal secretion. Our cases’ ACC were not functional. Hormone secretion is not a discriminating feature between benign and malignant adrenocortical masses. The silent clinical nature of nonfunctioning ACC results in poor outcomes, while the majority of patients present with locally advanced and/or metastatic disease. The work-up for adrenal masses must include determination of whether the mass is functioning or nonfunctioning and whether it is benign or malignant [1, 5].

Radiographic studies in the form of CT or magnetic resonance imaging (MRI) can help define the size of the mass and rule out metastases. Emerging evidence suggests that fluorodeoxyglucose positron emission tomography (FDG-PET) with CT is superior to CT alone [6]. However, FDG-PET/CT is still considered a complementary study and is not recommended for ACC work-up [6].

Large tumors of > 4 cm raise high clinical suspicion of malignancy, as in our cases, and favor radical adrenalectomy [7, 8]. To the best of our knowledge, the first of our two case reports describes one of the largest nonfunctioning ACCs reported in the literature in the past 10 years.

Despite the poor prognosis in this patient group, chemotherapy has a limited role in the treatment of ACC, and surgical resection has been shown to have the best outcomes [9, 10]. However, recent studies have shown a role for adjuvant chemotherapy in prolonging recurrence-free survival and overall survival [11]. There is no established duration of adjuvant chemotherapy.

Allolio *et al.* considered that complete surgical resection (Ro excision) offers the best chance for long-term survival in patients with stage I–III ACC, although these patients should be candidates for chemo-irradiation in order to increase disease-free survival [12]. In cases of recurrence, surgery should be considered a first-line option. However, mitotane has a valuable role in stage IV cases or in the presence of recurrent disseminated disease [12, 13].

Studies demonstrated mitotane’s associated toxicity and its ability in: (1) inhibition of adrenocortical steroid biosynthesis by inhibiting cholesterol side chain cleavage and 11 β-hydroxylation, and (2) induction of hepatic clearance affecting extra-adrenal disposition of cortisol [14]. Concerning the routine use of mitotane, there is a lot of controversy as partial response occurs for 5–30% of patients with ACC treated with this anticancer agent. High-risk patients are currently being treated for 5 years with mitotane.

Alternatively, in patients with proven lung metastasis, the published evidence suggests that en bloc excision of involved organs and pulmonary metastasectomy could improve overall survival.

Postoperative surveillance for recurrence should be performed every 3 months for the first 2 years and then every 6 months for 5 years.

Adrenal pseudocysts most commonly arise from hemorrhage within the adrenal gland, secondary to extreme stress, birth, trauma, surgery, or malignancy. The differential diagnosis of adrenal pseudocysts is from endothelial epithelial and parasitic cysts and rarely from mesothelial cysts, lymphangiomas, dermoid cysts, or cystic adrenal carcinomas.

Most pseudocysts on CT reveal well-demarcated, round or oval masses with fluid density with content of septa, blood, and soft tissue components. The wall of a pseudocyst seems to have occasional calcification on CT. MRI is pathognomonic for visualizing the complicated intracystic components [15].

Regarding surgical treatment, the open approach is preferable if the patient is symptomatic, the mass is > 6 cm, or there is a possibility of malignancy. Laparoscopic surgery is indicated for small tumors or pseudocysts provided there is no peri-adrenal infiltration and subsequent capsular disruption [15, 16].

## Conclusions

ACC is a rare and aggressive malignancy. Non-secretory adrenal masses are diagnosed late by a mass effect, metastatic disease, or found incidentally. The work-up for adrenal masses must include determination of whether the mass is functioning or nonfunctioning and whether it is benign or malignant.

We present the cases of two women who presented to our department after being evaluated for fatigue, mass effect, and epigastric discomfort. Both of them were diagnosed as having a nonfunctioning ACC and underwent open adrenalectomy. To the best of our knowledge, the first case’s nonfunctioning ACC is considered to be one of the biggest that has ever been described in the literature.
