# Correction to: Giant nonfunctioning adrenal tumors: two case reports and review of the literature

**DOI:** 10.1186/s13256-018-1932-4

**Published:** 2018-12-06

**Authors:** George Chatzoulis, Ioannis Passos, Dimitra-Rafailia Bakaloudi, Dimitrios Giannakidis, Alexandros Koumpoulas, Konstantinos Ioannidis, Ioannis Tsifountoudis, Dimitrios Pappas, Panagiotis Spyridopoulos

**Affiliations:** Department of Surgery, 424 General Military Hospital of Thessaloniki, Agiou Nikolaou 42, 55,132, Kalamaria, Thessaloniki, Greece

**Correction to: J Med Case Reports (2018) 12:335**

**https://doi.org/10.1186/s13256-018-1876-8**

In the publication of this article [1], there is an error in the Family Name and Given Name of the authors since these were interchanged.

The error:

Chatzoulis George, Passos Ioannis^*^, Bakaloudi Dimitra-Rafailia, Giannakidis Dimitrios, Koumpoulas Alexandros, Ioannidis Konstantinos, Tsifountoudis Ioannis, Pappas Dimitrios and Spyridopoulos Panagiotis

Should instead read:

George Chatzoulis, Ioannis Passos^*^, Dimitra-Rafailia Bakaloudi, Dimitrios Giannakidis, Alexandros Koumpoulas, Konstantinos Ioannidis, Ioannis Tsifountoudis, Dimitrios Pappas and Panagiotis Spyridopoulos

This has now been updated in the original article [1].
